# Designing Effective Interactions for Concordance around End-of-Life Care Decisions: Lessons from Hospice Admission Nurses

**DOI:** 10.3390/bs7020022

**Published:** 2017-04-18

**Authors:** Carey Candrian, Channing Tate, Kirsten Broadfoot, Alexandra Tsantes, Daniel Matlock, Jean Kutner

**Affiliations:** 1 Division of General Internal Medicine, Department of Medicine, University of Colorado School of Medicine, Aurora, CO 80045, USA; jean.kutner@ucdenver.edu; 2 The Adult and Child Consortium for Outcomes Research and Delivery Science, Department of Medicine, University of Colorado School of Medicine, Aurora, CO 80045, USA; Channing.Tate@ucdenver.edu (C.T.); daniel.matlock@ucdenver.edu (D.M.); 3 Center for Advancing Professional Excellence, Department of Medicine, University of Colorado School of Medicine, Aurora, CO 80045, USA; kirsten.broadfoot@ucdenver.edu; 4 The Denver Hospice, Denver, CO 80246, USA; alexandratsantes@gmail.com; 5 Division of Geriatric Medicine, Department of Medicine, University of Colorado School of Medicine, Aurora, CO 80045, USA; 6 VA Eastern Colorado Geriatric Research Education and Clinical Center, Denver, CO 80220, USA

**Keywords:** hospice decision-making, concordance, end-of-life communication

## Abstract

Near the end of life, hospice care reduces symptom-related distress and hospitalizations while improving caregiving outcomes. However, it takes time for a person to gain a sufficient understanding of hospice and decide to enroll. This decision is influenced by knowledge of hospice and its services, emotion and fear, cultural and religious beliefs, and an individual’s acceptance of diagnosis. Hospice admission interactions, a key influence in shaping decisions regarding hospice care, happen particularly late in the illness trajectory and are often complex, unpredictable, and highly variable. One goal of these interactions is ensuring patients and families have accurate and clear information about hospice care to facilitate informed decisions. So inconsistent are practices across hospices in consenting patients that a 2016 report from the Office of Inspector General (OIG) entitled “Hospices should improve their election statements and certifications of terminal illness” called for complete and accurate election statements to ensure that hospice patients and their caregivers can make informed decisions and understand the costs and benefits of choosing hospice care. Whether complete and accurate information at initial admission visits improves interactions and outcomes is unknown. Our recent qualitative work investigating interactions between patients, caregivers, and hospice nurses has uncovered diverse and often diverging stakeholder-specific expectations and perceptions which if not addressed can create discordance and inhibit decision-making. This paper focuses on better understanding the communication dynamics and practices involved in hospice admission interactions in order to design more effective interactions and support the mandate from the OIG to provide hospice patients and their caregivers with accurate and complete information. This clarity is particularly important when discussing the non-curative nature of hospice care, and the choice patients make to forego aggressive treatment measures when they enroll in hospice. In a literal sense, to enroll in hospice means to bring in support for end-of-life care. It means to identify the need for expertise around symptom management at end-of-life, and agree to having a care team come and manage someone’s physical, psychosocial, and/or spiritual needs. As with all care, hospice can be stopped if it is no longer considered appropriate. To uncover the communication tensions undergirding a hospice admission interaction, we use Street’s ecological theory of patient-centered communication to analyze a case exemplar of a hospice admission interaction. This analysis reveals diverse points of struggle within hospice decision-making processes around hospice care and the need for communication techniques that promote trust and acceptance of end-of-life care. Lessons learned from talking about hospice care can inform other quality initiatives around communication and informed decision-making in the context of advance care planning, palliative care, and end-of-life care.

## 1. Background

Near the end of life, receiving hospice care is associated with less distress, fewer hospitalizations, and improved caregiver outcomes [1,2,3,4]. However, the decision to enroll in hospice while dependent on an individual’s knowledge of hospice and its services is also heavily influenced by diverse emotions and fears, cultural and religious beliefs, and levels of acceptance around the diagnosis [5,6]. The more interactions an individual has with nurses, physicians, chaplains and social workers, family and friends, the greater their understanding of hospice. One key interaction in this chain of influence around hospice enrolment is the hospice admission consult [6]. While this unpredictable, nonstandard, and highly variable interaction often occurs late in the illness trajectory, it plays a significant role in shaping decisions regarding end-of-life care. Because hospices hold an obligation to provide accurate information to their patients (beneficiaries) about the hospice benefit, election statements and the clarity of information provided in admissions consults has been recently examined by the Office of Inspector General (OIG) [7].

The OIG 2016 Report, “Hospices should improve their election statements and certifications of terminal illness,” states that resolving disparities and vulnerabilities in election forms is crucial to ensuring patients and caregivers make informed hospice care decisions. It calls for hospices to provide complete and accurate information at the initial consult [7]. However, outside of a clear election statement, how else can we improve initial admission interactions? Our own qualitative pilot research on these complex consultations is guided by two objectives: (1) enhance clinician understandings of patient and caregiver expectations and needs around hospice admission conversations and (2) identify communication techniques to facilitate concordance, or a point of commonality between individuals’ preferences (what they want), concerns (what are they against), and circumstances (the facts of their life), that incorporates patients’ and caregivers’ perspectives. The pilot data highlights diverse and often diverging stakeholder-specific expectations and perceptions based on setting, situation, diagnosis, and patient, caregiver, and nurse perspectives. What becomes clear in both observation data and the analysis of in-depth interviews with hospice nurses, patients, and caregivers, is that participants are often on different pages when they enter these interactions, endangering concordance and requiring nuanced communicative efforts from first contact to establish trust and credibility with each other.

Not only do parties in the interaction carry with them diverse and often diverging interests and motivations, the interaction itself occurs in a stress-laden context. The hospice admissions nurse or social worker must balance the intersecting needs of patients often too ill and fatigued to participate, overburdened caregivers who fear that choosing hospice means giving up on loved ones, and referral sources or other healthcare providers anxious for a quick hospice transition. These tensions and competing needs necessitate clear, tailored communication during the consult [6]. Reaching concordance is critical for any type of informed decision-making around advance care planning and other end-of-life care conversations [8,9].

Hospice admission nurses in particular are at the forefront of facilitating quality interactions geared towards concordance around hospice care. To illustrate the nature of this interaction and address the research gap surrounding it, we present a detailed narrative case of a hospice admission interaction and critically analyze the communicative practices used by the hospice nurse and caregiver using the social ecological model. Of note, in the initial hospice consult, we talk generally about palliative care as a program of care that is appropriate when patients are facing advanced illness and have symptom management needs (or maybe just extra psychosocial support needs), but are still continuing treatment of their disease. On the other hand, we talk generally about hospice as meaning the patient is receiving symptom management support, but is not treating their disease. Moreover, palliative care programs are not standardized (or paid for) like hospice is, so palliative programs can look different depending on where individuals are receiving care. 

## 2. Analyzing Hospice Admissions from an Ecological Perspective

The primary goals of the hospice admission interaction are (1) to exchange information between provider, patient, and caregiver about health-related concerns; (2) make decisions about medical care; and (3) in the best of cases, establish or maintain a relationship characterized by rapport, trust, and respect [6]. As such, it is a dynamic, creative, and complex event. How the interaction unfolds depends on how participants select, adapt, and coordinate responses to accomplish their individual and mutual goals.

**Figure 1:**
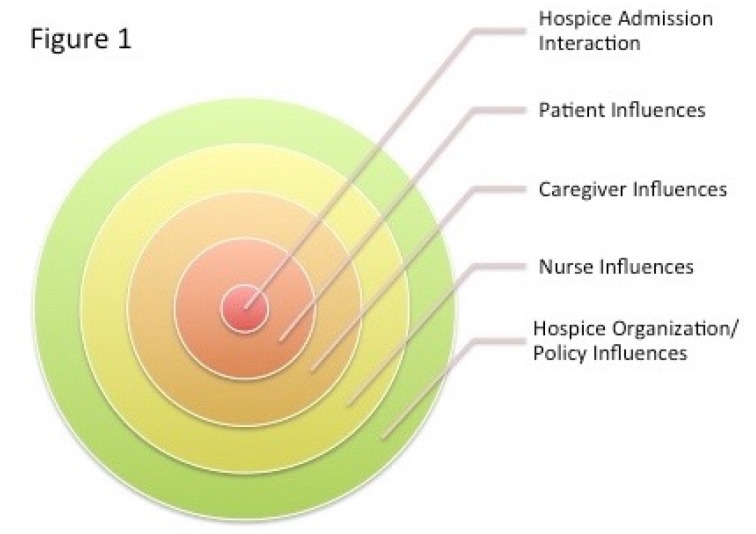
Ecological model of hospice admission interactions.

Street’s ecological theory of patient-centered communication focuses on the complex interplay between individual, relational, community, and societal influences on interactions around health [10]. This theoretical framework approaches the hospice admission interaction not as an isolated event, but as embedded within a number of contexts. In doing so, it enables an in-depth understanding of the range of factors that put pressure on individuals when making decisions (see Figure 1): 

*Interpersonal context*: includes predisposing influences of the provider, patient, and family (e.g., communication style, attitudes, beliefs, personality, and linguistic resources), as well as cognitive-affective influences of participants (e.g., perceptions, communicative strategies, and emotional state). 

*Political-legal context*: includes Medicaid/Medicare coverage and conversations around medication discontinuation and payment.

*Cultural context*: includes influences of ethnicity, socioeconomic status, philosophy of care, and religion.

*Organizational context*: includes standards of care, services offered, facility restrictions on where interaction takes place, hospice admission training, and hospice organization goals. 

Although the hospice admission encounter may be contextualized, influenced, and understood in a number of ways, the one within which the hospice admission encounter is most fundamentally embedded is the interpersonal context.

## 3. Research Setting and Sample

The project grew out of a long-standing community-academic partnership between a local hospice and university. Monthly agendas for this partnership meeting included an item titled, “what bugs you.” During a meeting in 2015, in response to this agenda item, the president of the hospice talked about the significant variability and non-standardization of hospice admission visits and the desire to have evidence, or a core set of best practices, surrounding admission visits so that patients and families who would like to benefit from hospice are able to do so while fully understanding what their decision to enroll means. The local hospice is a large, urban, non-profit organization that has served more than 70,000 patients and their families since its founding in 1978. It is the fifth longest-established hospice in the United States and remains a well-respected leader at local and national levels. Patient census reported 506 hospice patients and 214 palliative care patients under their care each day in 2014. In 2015, the numbers climbed to 532 and 227, respectively. Services are provided by teams of professionals and volunteers focused on individualized, integrative care in patient homes, skilled nursing facilities, or in their own Inpatient Care Center. No patient is ever turned away regardless of ability to pay. 

In 2015, the president of the hospice put the first author in contact with the admissions manager (A.T.) to talk about the project and the opportunity to “shadow” hospice admission nurses on their initial visits with patients and caregivers. The admissions manager developed a schedule that assigned specific days and times with different admission nurses and settings over a three-month period. Because admission visits were often scheduled the day before, the admissions manager would email the first author (C.C.) in the morning with the schedule for visits and text during the day as others were scheduled or cancelled. The schedule included home, nursing home facilities, and hospital room visits across an area in the Rocky Mountain region of the United States.

When visits were scheduled, A.T. would email C.C. and the assigned nurse with a brief overview of the visit including the patient’s age, diagnosis, and anything important to know before the visit (e.g., this was the patient’s second admission visit; do not use the word hospice; family is struggling with this decision); C.C. did not access medical charts. C.C. would then confirm a meeting place with the nurse before the interaction started so they could walk in together and as needed, be briefed about the patient, family situation, and project beforehand. The admission visits lasted between 30 min to 2 h. There were visits that had as little as three people present, and as many as nine, including the hospice nurse and C.C. To ensure a diverse sample, participants included: African Americans, Latino, undocumented, Russian, Caucasian, tribal, homeless, religious, non-religious, gay, straight, married, and widowed. Additionally, nurses that were shadowed were also diverse in terms of years at hospice and experience before coming to hospice. 

On site, C.C. was introduced to the patient and family as a researcher from the local university studying hospice communication to define best practices. In all cases, patient and/or caregivers verbally consented to researcher presence before starting. During these conversations, C.C. sat next to the nurse, or in some cases, wherever the family encouraged her to sit based on the arrangement of the room/setting. Ethnographic field notes were minimally taken to capture key words or phrases used during the interaction, and sometimes not at all depending on the situation. In these cases, C.C. would write them directly after the visit. Note taking was not considered obtrusive, as the admissions nurse also had a notebook and took notes during the conversation. C.C.’s role in these interactions was solely to observe, and patient and family interaction was limited to polite greetings and good-byes. 

At the end of each admission visit, and always dependent on the situation and comfort of participants, C.C. asked the patient and/or caregiver if they would be willing to answer five questions related to the visit. There were five instances where C.C. did not ask the participants given the perceived distress or known circumstances that would have impinged participation. For the other 20 visits, all who were asked to voluntarily participate agreed to so do. 

## 4. Data Collection and Analysis

C.C. conducted nonparticipant observations of hospice admission visits between hospice admission nurses and patients and their caregiver(s) over a three-month period during 2016–2017. The setting of these interactions varied from hospice home visits, skilled nursing facility visits, and hospital room visits. The resulting data set included 60 h of observation, including interviews with 15 caregivers, 6 patients, and 9 hospice admission nurses (*n* = 30). All interviews were audio-recorded. Because of the health of participants, circumstances around each visit and ethical considerations guiding every stage of this project, interviews lasted no longer than 15 min to respect the comfort of participants and the time with their loved ones, if present. Of the observed admission visits, 20 enrolled in hospice, 3 were undecided, and 2 declined hospice. 

C.C. also attended hospice admission monthly staff meetings where “defining best practices,” was a standing agenda item (*n* = 3). Before observations began, C.C. provided a short presentation at the staff meeting about the project: what was required of admission nurses, the goals of the observation and interviews, how results would be shared back with the organization, and answered any questions they had. None of the admission nurses chose to opt-out of participating. Ethnographic field notes of these meetings were written and typed single-spaced for a total of 250 pages (8–12 pages per visit). 

Interviews were transcribed verbatim and then analyzed alongside the ethnographic field notes using thematic discourse analysis in an inductive, data-driven approach. Each observed admission conversation was broken down and coded, allowing the researcher to establish a pattern by relating codes/categories to one another. The Colorado Multiple Institutional Review Board approved the study (protocol # 16-1897).

## 5. Ethical Considerations

This project would not be possible without a strong ethical foundation. Ethics have been incorporated from initial study design to the sharing of results. The skill of introspection and the ability to accept and process feedback regarding very personal aspects of this work are important attributes of any researcher doing end-of-life research [11]. The instrument in ethnographic qualitative research is a human instrument: the researcher as a whole person in the midst of a culture being studied. Therefore, a profound awareness and understanding of the nature of the constructed boundaries of one’s own identity and personal experience is critical to being an effective human instrument.

This project has specifically been guided by post-colonial ethics of accountability, context, truthfulness, and community. More specifically, through a felt need of endorsing other models of reflexivity, we have looked to others who have done similar work, like Ellingson, Broadfoot, Foster, Hirschmann, de la Garza, and Eisenberg [12,13,14,15,16], documenting the ethical tensions that arise and how they worked through them. An ethical concern and models of reflexivity extend the challenge of speaking for others, which is interrelated with the politics of representation and the crisis of legitimation around qualitative work [17,18]. In order to work with this tension and challenge, Alcoff encourages researchers to not speak for, but to speak with participants by engaging them in conversation and representing the writing in a way that is meaningful and accessible to the ones who have shared and created their stories at extremely fragile times in their lives [17]. In an effort to work through the challenges of representation, alternative forms of writing, such as narrative styles and case studies have been used to escape the constraints of traditional writing as well as respond to the ethical tensions of speaking for others in qualitative research. Further, pseudonyms have been used throughout this paper to protect participants’ identities.

For these reasons, we have chosen a case study exemplar for this paper, as a powerful example of the many issues associated with hospice admissions [19]. Although we have pages of observations and conversations documented in field notes (conversations were not audio taped, just the interviews), we chose this particular narrative because it is a comprehensive exemplar of our larger data set and it effectively highlights the ecological nature of these interactions. Through this lens, our analysis highlights critical communicative acts and turning points and demonstrates the inherent complexities of hospice admission interactions. 

Establishing concordance is necessary to coordinate communication and decision-making, as it sets the tone for all future interactions around hospice [8]. Pilot data illustrates that what unfolds in these interactions ultimately depends on the communicative actions that emerge directly from interactants’ goals, linguistic skills, perceptions, emotions, and knowledge, as well as the constraints and opportunities created by the responses of others (i.e., caregiver, patient, and hospice nurse). Using an ecological lens to view these interactions shifts analytical attention beyond focusing on relationships between providers and patients and the various outcomes resulting from the interaction (e.g., satisfaction with care, commitment to treatment, health improvement) to describing concrete processes within the admission interaction that affect communicative action [8,20].

## 6. Real Case Exemplar: “I Don’t Want to Go Back and See Her, Just Tell Me If It’s Time for My Mom to Start Hospice”

### 6.1. Background and Context to the Interaction

It is cold but sunny and glass doors open onto a dark lit room in the Alzheimer’s Care Center. The facility walls are decked out in holiday decorations, and Christmas music can be heard playing in the background. James, the hospice admission nurse and I (C.C.) have an appointment to meet with the patient’s daughter, but first we need to look through her record and talk with her nurse. There is no one in the halls as we enter, save a patient trying to exit. It smells of feces, urine, and artificial cleaning products. It is hard to breathe. Several residents sit in their wheelchairs at the entryway of their rooms, lined up as though they are waiting for a delivery. Some look up as we walk by, while others are sleeping with their heads cocked to one side. Other residents meander through the hall, scuffing their feet across the carpet to move their wheelchairs. The smell gets stronger the further we go. We pass the “living room” filled with about 15 residents gathered in a semi-circle around the TV in their wheelchairs. Every head is bowed with eyes closed.

We arrive at the nurse’s station and, upon request, receive a large blue binder containing the patient’s record. As we open it, another resident approaches the desk. James greets her and asks if he can help, and she says there is a lot he can help with before walking away. A patient yells at a nurse as they walk out of the dining room because she is holding his arm (to prevent a fall). I hear a conversation between another nurse and patient involving a nail gun, as the patient complains that it sounds like someone has been kicking the wall. “Lunch is in an hour”, another nurse says to a patient sitting in the hall as she walks by a nursing aide wheeling a woman backwards into the shower room.

### 6.2. Notes from Hospice Organization to the Admissions Nurse

Patient and family are undecided on whether to enroll patient into hospice services or not. Dr. Hanks has expressed to them that he does not think patient is hospice appropriate at this time, but is open to reconsidering this based on hospice’s evaluation. Family is also wanting more information and an evaluation to determine whether patient is appropriate or not. Daughter has many concerns about admitting patient to hospice, and several questions on why or why not her mother would qualify. Daughter would like hospice nurse to lay eyes on patient, then meet with her in the lobby after. 

### 6.3. The Patient

The patient’s nurse tells us that Judy is a “very pleasant 93-year-old. She has congestive heart failure (CHF) and had a change of condition a few days ago: fluid in her lungs. Her appetite is good and she is ambulatory. She has a little pain, does not talk much, just general facial expressions. She had antibiotics to help treat the fluid and has gotten a little better”. James gets her medications and confirms that there is only one medication with the nurse. He asks about a note in the record that outlines the family’s request to not call the house before 8 am for non-urgent matters and asks how this affects the nurse’s interactions with them. There does not seem to be any concern, but the nurse shares that the family used to come every week but that lately no one has been around. James closes the binder, puts it back on the shelf, and signals me to follow. We make our way back up to the lobby to meet Emily, Judy’s daughter. Four patients in wheelchairs are lined up looking at the front door. It seems they are going out to Applebee’s. They wait for a staff member to pull their transport around, only to find that it is not operational. They are devastated by this news.

### 6.4. Interaction with Emily the Family Caregiver (Daughter)

We find Emily in the lobby after she dropped a stack of clothes at the receptionist’s desk. We introduce ourselves and head somewhere to talk.

J: “Before I give you a bunch of information, can you tell me your understanding of hospice?”

E: “I understand it as two levels of care. Palliative being less hands-on, and hospice being more involved.”

(James takes a pause and elaborates on the meaning of both, underscoring the additional support of hospice and the benefits of both for her mother.)

J: “I saw your mom very briefly earlier today and she is doing well. She got up by herself and walked about the room. She’s continent.”

E: “She’s continent? She has been in a diaper every time I have seen her.”

J: “Sometimes around here they put diapers on almost like a security blanket so the patients can be relaxed if they have an accident or can’t get up quickly enough to the restroom. We can go see her after we finish our conversation.”

E: “I don’t want to go back.”

[*Turning point #1*: In this moment, James is having this conversation without the patient in the same room because the daughter does not want to go back to see her. One of her expectations for James today is to evaluate her mother, but not being able to see her while speaking with the daughter makes providing recommendations challenging for James. While James saw her very briefly that morning, he is not sure the last time the daughter has seen her. Thoughtfully and openly, James knows they will not be going back, and also knows the daughter is not yet ready to hear details about her mother’s health condition, so James turns the conversation.]

J: “What are your goals today?”

E: “We want an honest assessment of where she is to determine if we should enroll in palliative or hospice care. You are the experts. We are leaning more towards palliative care, but we just don’t know. Is she eligible for hospice? Would her doctor support that decision?”

[*Turning point #2*: James has already asked and suggested they go back so he is able to provide what the daughter wants: an honest assessment. However, having the conversation in the mother’s room is not an option. Another layer of complexity is added when she introduces two competing perspectives, stating that James and hospice are “the experts” before quickly asking, “would her doctor support that decision?” It is unclear who carries more credibility. This makes it difficult for James to know what role he needs to play. In response to the question “What are your goals?” Emily redirects decision-making responsibility to James by effectively stating, “tell me what to do.”]

J: “Yes, he said he would support that decision.”

E: “I’m confused—I thought he didn’t feel she was appropriate?”’

J: “Initially, Dr. Hanks said he didn’t think she was appropriate for hospice, but in the chart from November it states he would support the decision to enroll in hospice if we felt she met criteria, which I feel she does. After seeing your mother, I think she can benefit from palliative or hospice care, and I have information and paperwork on both options today. But this decision is ultimately for you and your family to make. Do you know what she wants?”

E: “No, I mean she can’t communicate.”

[*Turning point #3*: Emily is navigating some inconsistencies—the doctor was originally unclear about her mother being hospice appropriate, but now it appears that she is. James asks another question to try and elicit the degree to which they have discussed her mother’s preferences around end-of-life care options.]

J: “Did you talk with her before about what she would want?”

E: “Not really. But I think she’s ready for palliative care and until the doctors feel absolutely certain she is ready for hospice, we will go from there. Is that reasonable?”

J: “Yes, absolutely.”

E: “Can you re-evaluate her in 90 days? It’s risky, I know, since she could go downhill quickly and maybe need more help.”

J: “It’s a very difficult decision, and we want to help you in whatever way we can. We can fill out both paperwork, and you can think more about it and let us know within a few days.”

E: “Do you think she is ready for hospice?”

J: “I think she is, but the decision to enroll is really up to you. She is eligible and would certainly have more eyes on her with a larger care team.”

E: “Would she have more interaction and would your team interact with the team here okay?”

J: “Yes, she would have more frequent visits on our hospice program, as our palliative team serves more in a consultative capacity. But know that both programs provide an extra layer of support to our patients. Hospice also works hard to provide support to you as her family member…we know this is a challenging time.”

E: “I don’t know what to do. This is really hard and you’re not giving me any guidance!” (Emily gives her first brief smile.)

[*Turning point #4*: James has outlined several options for Emily about going forward (signing both papers and making a decision later), but during this exchange it is clear that Emily is struggling with what to do and keeps asking reassuring and guidance-seeking questions to James. In James’s mind, he is helping her: asking questions about goals of care, asking what the mother would want, supporting her concerns, clarifying her understanding of hospice and palliative care, and leaving the decision very open. Ultimately, James is encouraging Emily to weigh the options. At this point in the interaction, Emily feels stuck and fearful of making the wrong decision. James astutely recognizes that not all family members feel comfortable making decisions or choosing between options, and that some interactions require more direct, less open-ended communication to help with the decision-making.]

J: “We just want you to be prepared as possible should things get worse, and we want to be able to best support you all. So often patients and families wait until they are close to dying before enrolling in these services, whereas signing up earlier could really benefit not only your mom, but you and your siblings as well. If I could make a recommendation, I would encourage you to sign your mom up for palliative care today and then we can move her onto hospice services when you feel more comfortable with that transition. If you change your mind, just give us a call; these decisions are not set in stone. Your palliative team will also help you understand when the time is right for a transition to hospice.”

(Emily takes a deep breath, uncrosses her arms, and leans in towards the table.)

E: “You know, my father died 10 years ago. My mom insisted he get cancer treatment until the day he died while living at home. It was a Saturday and my mother wanted us to haul him to the hospital for another treatment. He was so sick and so weak. And my family and I finally intervened and told my mother no. He died that following Monday.” (pauses) “I want hospice. Let’s go directly to hospice. I don’t want to have her be on hospice for 4–5 years, but I also don’t want to ignore something she might really need and benefit from. This is so hard.”

[*Turning point #5*: James’s patience and use of silence allows Emily to open up and tell this story about her father, which has had a significant impact on how she is navigating this decision for her mother. Moreover, as James plays the role of expert in this moment, it opens things up for Emily to say something different (e.g., remembering her experience with her father). James’s silence also allows Emily to come back and answer his first question about goals of care.]

E: “I want her as comfortable as possible and free of pain, with an extra set of eyes.” (Emily’s eyes fill with tears. James pulls out the paperwork.)

J: “Just remember that no matter what you decide, you can always change your mind at any point.”

E: “You can re-evaluate her at 90 days, right? This is so hard.”

J: “Yes, we can, and I will put that in the notes.”

(James starts to go through the enrollment paperwork Emily needs to sign to get her mom enrolled in hospice. Emily has no questions.) 

[*Turning point #6*: Organizationally, this is an effective interaction because there is a plan in place: to sign up for hospice care with the understanding that if it is not the right fit, Emily can discontinue services at any time. However, psychosocially, did Emily get the help she needed? This is the more challenging question to answer. Is it James’s fault if she did not? Many times, families cannot reach a decision for a number of reasons—societal norms around the meaning they have assigned to hospice, deep family dynamics that still need to be addressed and dealt with, acceptance of the illness, acceptance of the reality of caregiving needs, uncertainty what their loved one wants, fear of financial burden keeping their loved one home, even if it is what they want, and so on. The interaction comes to a close in the next lines when James is filling out paperwork and having Emily sign the consent forms, when he notices Emily’s birthday on the power of attorney form.]

J: “Your birthday is coming up!”

E: “And so is my mom’s.”

J: “Are you having a party for her?”

E: “No, actually. I will be in Chicago.

(Judy’s nurse Katie comes in to have Emily sign a paper.)

E: “Hi, Katie, how is she doing?”

E: “Is she still on oxygen?”

K: “No, just as needed.”

E: “I left some clothes for her at the front. Can you take them back to her?”

K: “Sure.”

(Emily signs the last document. She takes her copies, grabs her bag.)

E: “Thank you, and I will talk with you soon, ok? I would like to know more about that bereavement program for children you spoke of because my mother’s granddaughter has a lot of emotional issues and would benefit from that support. Can you put that in the notes?”

J: “You got it. Thanks for coming, Emily.”

The hospice admission encounter, like the one outlined above, reveals a variety of reoccurring patterns of communication and struggle. Looking at the narrative, there are clear communication challenges, as well as opportunities to assess each unique situation, connect, clarify, and create new meanings. Identified turning points illustrate how the outcomes of these interactions ultimately depends on the communicative actions of participants and their goals, linguistic skills, perceptions, emotions, and knowledge, as well as the constraints and opportunities created by the responses of others (i.e., caregiver, patient, and hospice nurse). Examining this interaction through an ecological lens, the following points become apparent: 

*Interpersonally*: In not wanting Emily to feel forced into a decision, James initially struggles to provide a recommendation for one service over the other. Emily struggles to understand why James is not playing the role of expert and telling Emily what she should do. On a personal level, James struggles to understand why Emily does not want to go back and see her mother, and wonders what dynamics are at play. Emily is struggling to accept the new normal of her mother, including not wanting the same thing that happened to her father to happen to her mother. Emily is overwhelmed being her mother’s primary caregiver. 

*Organizationally*: Entering skilled nursing facilities to talk about hospice adds an extra layer of complexity to the encounter, as hospice nurses navigate the organization’s controls and rules, gaining access, finding the chart, and finding the patient’s nurse prior to starting the interaction. James’s experience working in skilled nursing facilities (and not always having a positive experience) as well as the training he has received at his particular organization regarding admissions conversations and communication strategies also shapes how he thinks and feels during admissions interactions and decisions. Emily’s presence inside the skilled nursing facility (and her wishes to not go past the front door) also influences the interaction. Furthermore, this is James’s second of four visits for the day. Visits can last between 30 min and 2 h, and often James does not have a lot of time between cases to debrief from the last one and prepare for the next.

*Culturally*: Emily and James navigate several cultural subtleties in their conversation. Broadly, both dealt with the culture of the nursing facility where the conversation took place. The cultural tensions of nursing home care are often dictated by insurance reimbursements and staffing shortfalls. These challenges frequently create a culture of apathy or indifference, which is difficult to manage. Further, the culture of the nursing home directly influences the ability of hospice to enter the facility and provide care. Emily, a retired US Parks Worker, entered this interaction looking for guidance and for someone to tell her what choice to make. She viewed James as the medical expert with an exclusive knowledge base who could quickly make the decision for her. The term “expert,” is used by groups and individuals to legitimize claims of expertise or competence. As a term and ‘position’ in society, the ‘expert’ is also able to influence how people understand and act in social realms and their world. 

This is a common paradigm in the culture of medicine in which paternalism and physician choice in medical decisions is the longstanding cultural norm [21]. As a hospice admissions nurse, James comes from a culture of information giving and decisional autonomy. A cultural tension exists in whether it is appropriate for James to exercise some paternalism and influence Emily’s decision as the expert or let her own cultural values and beliefs guide the discussion. Ultimately, Emily reflecting on her experience with her father allowed her to clarify what decision was best for her mother and family. Thus, the interaction and its decisions rest on the intersection of two forms of expertise and the struggle over who should determine next steps. Emily defers decision-making to whom she perceives to be the only expert (as verified by society and training)—James. James, however, wishes the decision-making would be guided by the person who has lived experience and is therefore expert—Emily.

What we learned from analyzing this interaction ecologically:

- *First, participants play multiple roles*: Hospice nurses are asked to play many different roles at any given moment (e.g., medical professional, social worker, therapist, end-of-life-care expert, facilitator, advisor, educator, etc.). It is important to have these roles defined and understood from the outset and re-confirmed throughout the interaction.
- *Second, participant goals vary*: Patient and caregiver goals can vary, change, and evolve visit-to-visit. Clarifying the goal of the interaction enhances the likelihood of establishing trust at the beginning of the encounter and reaching concordance.
- *Third, understanding intersection(s) is key to adaptation*: Understanding that roles and goals may come into opposition, but finding intersections where concordance, or points of commonality between people’s purposes, concerns, and circumstances exists and remaining focused and adaptive leads to better shared decision-making. Finding intersections quickly and returning to them frequently helps discern between what patients/caregivers want, what they do not want, and what role the nurse must play to help the patient and family achieve those goals in light of their unique circumstances.

Although we agree with the assertion in the OIG report that patients require complete and accurate information about the hospice benefit to make an informed decision, we have shown the impracticality of one standardized method of hospice enrollment. The competing demands and expectations of the patient, family, and provider all need to be tempered to maximize the effectiveness of these interactions. Analyzing hospice admission interactions through an ecological lens demonstrates how the OIG’s mandate for clear election statements for informed decision-making is tempered and mediated by the complexity and heterogeneity of each individual hospice admission interaction. Moreover, this case study demonstrates that achieving concordance and informed consent is far more nuanced than verbiage on election forms. It demands real-time interpretation of a patient’s goals of care, patience, adaptability, and awareness of the interpersonal, organizational, and culture tensions that impact effective communication and interaction design. 

## 7. Conclusions

Patients and families enter into hospice admission interactions with varying degrees of comfort and knowledge, having to make a significant decision at a time when their loved one is extremely ill and the family is often very fatigued. This is deep and profoundly difficult caring work for admission nurses, who often conduct multiple consults a day and are responsible for explaining the hospice benefit, completing consents and advance directives, holding a goals of care conversation, and coordinating care of each patient. Analyzing this interaction reveals the different spaces patients, families, and nurses can inhabit, and why finding concordance from the outset is integral to facilitating a meaningful interaction.

The critical function of communication studies is to improve dialogue and decision-making processes. In palliative and hospice care interactions, understanding the impact of particular communicative practices on patients and families in the extremely vulnerable terrain of advanced illness requires detailed, scientific, and theory-driven explanations and constructions of alternative meaning and discourses [6,8,10,20]. Applying Street’s ecological theory of patient-centered communication to these interactions allows us to see how different communicative practices in real time can impact the ability to reach concordance and shared decision-making. A qualitative methodological approach to these interactions uncovers the complexities of discourse around the end of life and the nuances of meaning surrounding illness and care options. How individuals experience hospice, and the ways in which social, political, and cultural contexts impinge upon parts of the hospice admission interaction further contribute to these complexities. Advancing the rigor of studies of communication in hospice care settings using a qualitative methodological approach can illuminate the communicative techniques and practices necessary to improve decision-making, as well as evaluate the causes and effects of meaningful communication in improving care [22]. Developing communication guidelines built around a sensitized, rather than standardized attitude, should be an immediate next step.
